# What’s behind a P600? Integration Operations during Irony Processing

**DOI:** 10.1371/journal.pone.0066839

**Published:** 2013-06-24

**Authors:** Nicola Spotorno, Anne Cheylus, Jean-Baptiste Van Der Henst, Ira A. Noveck

**Affiliations:** 1 Centre National de la Recherche Scientifique, Laboratoire Langage, Cerveau et Cognition (L2C2), Université de Lyon, Bron, France; 2 Centre de recherche français à Jérusalem (CRFJ), Jérusalem, Israel; University Of Cambridge, United Kingdom

## Abstract

The combined knowledge of word meanings and grammatical rules does not allow a listener to grasp the intended meaning of a speaker’s utterance. Pragmatic inferences on the part of the listener are also required. The present work focuses on the processing of ironic utterances (imagine a slow day being described as “really productive”) because these clearly require the listener to go beyond the linguistic code. Such utterances are advantageous experimentally because they can serve as their own controls in the form of literal sentences (now imagine an active day being described as “really productive”) as we employ techniques from electrophysiology (EEG). Importantly, the results confirm previous ERP findings showing that irony processing elicits an enhancement of the P600 component (Regel et al., 2011). More original are the findings drawn from Time Frequency Analysis (TFA) and especially the increase of power in the gamma band in the 280–400 time-window, which points to an integration among different streams of information relatively early in the comprehension of an irony. This represents a departure from traditional accounts of language processing which generally view pragmatic inferences as late-arriving. We propose that these results indicate that unification operations between the linguistic code and contextual information play a critical role throughout the course of irony processing and earlier than previously thought.

## Introduction

Imagine two fishermen who spend a day together and fail to make a single catch. When one fisherman says (1) to the other, he is clearly being ironic.

1. “This day has been really productive!”

In order to understand the irony the second fisherman needs to go beyond the linguistic code and understand the speaker’s intent [1], [2]. That is, while the semantic meaning of (1) is determined by its truth conditions (see e.g., [3]), understanding the speaker’s intended meaning requires the integration of contextual factors as well as “mindreading.” Understanding (1) involves going beyond what is conveyed linguistically. This amounts to a distinction between semantics (which concerns the truth-conditional meaning of the sentence uttered; see e.g., [3]) and pragmatics (which investigates the interaction between the sentence and contextual factors that allows the listener to grasp the speaker’s intended meaning; see e.g., [2], [4]–[6]) and the starting point of our analyses here.

According to Gricean and post-Gricean perspectives, part of the process of interpreting utterances is to understand the speaker’s intention. In other words, interlocutors in every conversation are intentional agents who are moved by beliefs and desires and who try to share, impose or suggest parts of their inner world. In (1), the speaker’s intended meaning includes communicating his disappointment in their day. It also follows that one ought to find evidence that listeners comprehend the intended message as they integrate the linguistic code with context. It is generally accepted that the interpretation of others’ thoughts and intentions is attributable to Theory of Mind (ToM).

Indeed, an fMRI study on irony processing by our group [7] has recently revealed that an ironic utterance in a brief story activates the Theory of Mind (ToM) network to a greater extent (see [8] for a description of the ToM network based on an extensive meta-analysis) than the same utterance whose interpretation is literal (which can be accomplished through control items having slightly modified contexts). Furthermore, that study revealed an increase of functional connectivity between language and ToM networks during irony processing; this interaction is arguably related with integration processing during the comprehension of an ironic utterance (see [7] for an extensive explanation of these results) Therefore, it has been demonstrated that the ability to integrate different streams of information – mainly the linguistic code and contextual information – is critical to getting at the complete meaning of an ironic remark.

As is well known, fMRI techniques do not allow for high temporal resolution, which prevents one from drawing strong conclusions about pragmatic inference-making processes *while* they are occurring on-line. However, in order to better address temporal concerns, several researchers have conducted ERP studies on pragmatic phenomena, including irony (e.g., [9]–[13]). This is what we turn to here.

### Pragmatic Inferences and ERP Components

The ERP components that have received the most attention in the investigation of pragmatic inference making are the N400 and the P600. While the N400 has historically been an index of semantic/world knowledge violations which occur at the sentence level (as shown in the well-known example, “He spread the warm bread with socks” [14]), it has also been shown that it more generally reflects *inconsistencies* between the linguistic code and the provided context (e.g., [15]–[17]). For example, Nieuwland and van Berkum [15] employed fictional contexts as they showed how an expression that is inconsistent with its immediate discourse context – even if it fits with one’s general knowledge – can elicit an enhancement of the N400. To give one specific example, they presented a story in which a peanut was as an animated character and, within this context, the utterance “The peanut was salted” elicited an enhancement of the N400 with respect to the utterance “The peanut was in love.”

The P600 was originally considered to be an index of syntactic violations (e.g., a violation of phrase structure, [18]) but it has since become associated with a broad range of linguistic expressions. For example, P600’s have been reported with respect to sentences that have semantic (reversal) anomalies (e.g., “The cat that fled from the mice”, [19]) and semantic (illusory) anomalies in a discourse context (e.g., “How many animals of each sort did Moses put on the Ark?” (e.g., [20]). The P600/Late positivity effect is also one of the most recurring outcomes from studies on pragmatic phenomena. For example, indirect requests (see for example, [21]), metaphors (see e.g., [9]) and ironies (see e.g., [11]) elicit an increase of the P600 component. However, considering the broad range of linguistic phenomena that can prompt a P600, it is hard to come up with a functional interpretation of the P600, even in the pragmatic domain, other than to say that these phenomena usually involve a mismatch between the literal meaning of an utterance and the message it conveys (e.g., when one says “John is a shark” it does not mean that John is a real shark; likewise, when (1) is used ironically it is meant to indicate that the day was very unproductive). A role for inconsistency is coherent with one of the major interpretations of the P600 effect, viz. an increase in the P600 correlates with an increase in sentence integration processes (for reviews see, for example, [22], [23]).

Given recent methodological advances, viz. the development of Time Frequency Analysis (TFA), it is clear that electrophysiological studies of language can now go beyond identifying ERP components and determining what may characterize them. Investigating the oscillatory dynamics of the brain with respect to language comprehension has the potential to identify, and in a fine-grained manner, how different streams of information are integrated in language comprehension and how different cognitive resources contribute to this process. In the current work, we follow a proposal from Bastiaansen (e.g., [24]) who assumes that two different cognitive processes, namely memory retrieval operations and unification operations, are among the most central to language comprehension (see Hagoort’s work e.g., [25] for a more detailed elaboration of this assumption) and that these link to oscillation dynamics. More specifically, memory operations – which refer to activities related to the retrieval and encoding of information – can be revealed through the theta and alpha bands while unification operations – which refer to the integration of two or more cognitive processes necessitated by a stimulus – can be detected through the oscillations in the beta and gamma bands. As will become clear, we will eventually focus on unification operations. However, before describing the link between brain oscillations and language processing in more detail, let us introduce a couple of crucial notions about Time Frequency Analysis.

### Brain Oscillations

TFA allows one to investigate the patterns of synchronization and desynchronization of neuronal activity related to the coupling and uncoupling of functional networks in the brain (see e.g., [26]–[29]). Elements pertaining to the same functional network are identifiable as such by virtue of the fact that they fire synchronously at a given frequency. One of the main features of the oscillations is that they are ongoing phenomena that occur even in the absence of any experimental task. As a result, the phase of the oscillation at the time of occurrence of the event is variable. Therefore, one can say that any change in oscillatory activity that is related to an experimental event is time-locked to this event, but not necessarily phase-locked to the event (on the contrary, ERPs are phase-locked responses). However, the experimental stimuli modulate the oscillatory activity and so event-related but non-phase-related responses may be meaningfully related to an event such as language comprehension. Considering that synchronization/desynchronization reflects the coupling/decoupling of neural networks, it can be argued that these oscillatory EEG responses provide a window into the functional network dynamics of the brain (see for example [24]).

#### Brain oscillations in language comprehension

Different aspects of language comprehension have been associated with several different frequency bands, namely theta (4–7 Hz), alpha (8–12 Hz), lower beta (13–18 Hz) and gamma (above 30 Hz). Synchronization in the theta band (synchronization is generally defined as the increase of power in a frequency band) has been associated with both the retrieval of lexical information and the encoding of new information into episodic memory (e.g. [30]–[32]). For example, Klimesch [31] claims that the increase of power in the theta band reflects the establishment of a “memory trace” during language processing. Desynchronization in the alpha band has also been associated with memory retrieval operations during language processing (e.g., [33]–[35]). Klimesch and colleagues have noted that desynchronization in the upper alpha band (broadly 10–12 Hz) positively correlates with semantic long-term memory performance (for a review see [36]). In contrast, the increase of power in the alpha band has traditionally been associated with cortical idling, but recent approaches consider the role of alpha oscillations to be related to both functional inhibition (see [37] for a review) and cortical excitability [38]. This is why it has been argued that desynchronization in the alpha band is associated with the engagement of source regions in a process while synchronization reflects the neutralization of task-irrelevant regions [37].

Turning to unification operations with respect to language processing, it has been found that the lower beta frequency range (13–18 Hz) is sensitive to the syntactic complexity of the stimuli [35] while several researchers found that gamma power correlates with semantic/world knowledge complexity of processing stimuli (e.g., [39]–[41]). For example, Hagoort et al. [39] presented subjects with three versions of the same sentence differing only in one adjective (3a–c):

1. “The Dutch trains are *yellow* and very crowded.”
2. “The Dutch trains are *white* and very crowded.”
3. “The Dutch trains are *sour* and very crowded.”

Since it is common knowledge to participants in the Netherlands that Dutch trains are yellow, the sentence in (3b) is incongruent with world knowledge, despite the fact that a train can obviously be white. In contrast, (3c) carries a stronger/semantic violation. This study showed that incorrect but plausible utterances (e.g., 3b) prompted an increase of power in the gamma band when compared to correct utterances (e.g., 3a) while the contrast between implausible utterances (e.g., 3c) and correct ones does not. These data suggest that gamma oscillations reflect unification processes in the semantic domain while also showing that time frequency analyses can make distinctions – between world knowledge violations and semantic violations – that cannot be detected by ERP analyses; note that both kinds of violation elicit an increase of the N400 component.

In addition, a study by van den Brink et al. [42] has shown that the integration of “semantic” and “social” information in the linguistic context affects different frequency bands. In their study, lexical semantic violations elicited an increase in the theta range across all participants, but only individuals with an empathic-driven cognitive style (as revealed by a psychological test) showed a larger increase of power in the gamma band in the presence of a speaker’s identity violations (e.g. when a sentence such as “I cannot sleep without my teddy bear” is uttered by an adult-sounding voice).

This short and partial review of the literature suggests that the analysis of brain oscillations can provide interesting insights into language comprehension and that TFA can reveal subtle distinctions that cannot be captured by ERP analyses alone. As Bastiaansen has noted, however, “experimental research into the oscillatory neuronal dynamics of unification operations have concentrated on semantic and syntactic unification only” ([24], page 26). Given that understanding a speaker’s meaning requires one to go beyond semantics and syntax, it would be worthwhile to focus on the oscillatory dynamics that is occasioned by pragmatic processes. That is why the present work extends the investigation of “unification operations” so that it includes the pragmatic dimension of language processing.

### The Present Study

Both theoretical accounts (e.g., [43]–[45]) and experimental studies (e.g., [7], [11]) assume that irony is a prototypical case of pragmatic inference making in which the processing of the linguistic code does not itself guarantee the comprehension of the utterance. Listeners need to integrate the linguistic code with the context in a manner that ultimately reveals the speaker’s intention. The electrophysiology literature on pragmatic processing, briefly summarized above, indicates that the most reliable cue of such integration processes (or unification operations) are the P600 and the gamma band. Therefore, in the present paper we will concentrate our attention of these markers. However, it is likely that the unification operations also require an extensive engagement of cognitive resources and so we will take a closer look at the increase or decrease of power in the alpha band in the likely event that these become evident.

It is relevant to point out that current debates on irony processing are dominated by three accounts that are, for the most part, focused on the (presumed) impact that literal meanings have on the figurative interpretation of an ironic utterance. The earliest comes from what is often called the Standard Pragmatic Model (SPM), which represents psychology’s best effort to create a processing model based on Paul Grice’s theoretical account of communication [1], [43]), according to which figurative utterances are departures from a norm of literal truthfulness. Irony is said to be understood because the speaker is blatantly violating a maxim of quality (“Do not say what you believe to be false”) which then triggers implicatures that can be calculated from the literal meaning of the sentence. Implicitly, according to these SPM accounts, figurative readings of an utterance ought to come with extra processing costs because readers need to detect violations and come up with richer interpretations, which would not be the case for literal readings of the same utterances.In reaction to the so-called SPM, Raymond Gibbs pointed to a host of figurative cases, including irony (and especially sarcasm), that provide readers with an enriched pragmatic reading without requiring extra processing (time) when compared to literal readings of the same utterances. Central to Gibbs’s account is the assumption that a figurative interpretation is constructed “directly” by the early integration of lexical and contextual information. In his own words, ‘‘People need not first analyze the literal, pragmatic-free meaning of an utterance before determining its figurative, implicated interpretation” ([46], 421).

One of the most influential accounts, Giora’s Graded Salience Hypothesis ([47], [48]), seeks to find an equilibrium between these two extremes. This hypothesis emphasizes that the most salient meaning of an ironic utterance is the one that is accessed first regardless of whether it is literal or figurative (e.g., ironic) and that, furthermore, the salient meaning of a figurative remark is the one that is encoded in the mental lexicon of the audience. Salience is defined as a function of different features such as familiarity, conventionality, frequency and prototypicality. Therefore, if the figurative meaning of an ironic utterance is encoded in the lexicon it will be processed first; in cases where less familiar (and then less salient) ironies are used, more inferential steps – and thus more cognitive effort – will be necessary for arriving at the intended interpretation. Much recent work presents supporting evidence for the Graded Salience Hypothesis [47]–[50] which sets itself up as being in opposition to the Direct Access view.

As far as we know there are five EEG studies on irony [10], [11], [51]–[53] and the most recurrent finding is that ironical sentences elicit P600/late positivity components that are larger than those prompted by control (literal) sentences. As reported above, this result is in line with a general tendency reported in the ERP literature with respect to pragmatic phenomena. Nevertheless, it is difficult to come up with a functional interpretation of the P600 because a wide range of linguistic phenomena elicit enhancement of this component. Another limitation of ERP studies on irony is that they are focused on the three psycholinguistic accounts of irony described above. In the most recent study [11], for example, the authors aimed to determine the extent to which their results verify the predictions of the Standard Pragmatic Model, the Direct Access View and the Graded Salience Hypothesis. The data revealed just a partial match with both the Standard Pragmatic Model and the Graded Salience Hypothesis. This led Regel et al. to call for a revision of the psycholinguistic models of figurative language comprehension. By considering a wider array of theoretical approaches, one could be in a better position to understand the multiple processes that are generated by irony.

Rather than focus on the features of irony processing from the point of view of pragmatic theories, the present EEG study focuses on the integration of different streams of information while paying particular attention to those that can be described as uniquely pragmatic. Through both ERP analysis and TFA, it is our goal to have a clearer picture of critical moments of information integration in irony comprehension. By applying TFA to irony processing this approach promises to highlight how two critical aspects of comprehension – the linguistic code and context – combine.

## Materials and Methods

### Participants

Twenty healthy participants, who were students from the Université de Lyon, participated in the study. All participants (whose mean age was 23; no minors have participated to the study) were native French speakers, were right-handed and reported to have normal vision and no history of mental illness. In accordance with the principles of the Declaration of Helsinki, the study was approved by the Ethical Committee CPP Sud-Est II in Lyon. All participants gave their written informed consent prior to the beginning of the experimental session.

### Materials

Sixty story-frameworks were created (in French) that led to a target sentence that could be interpreted either as ironic or as literal as a function of a minor modification made to prior context. For example, a story about two opera singers could describe a negative event (e.g., a terrible performance) or a positive context (e.g., an impressive performance) and thus give the utterance in (4) an *Ironic* or *Literal* interpretation, respectively:

1. “Tonight, we gave a superb performance.”

Otherwise, the introductory sentences and the wrap-up sentences of any given framework were the same for both conditions. The structure of the stories was the same as the one employed in Spotorno et al. [7] and thus contained the following six features:

First, all stories were seven lines long, each having a maximum length of 91 characters (spaces included) in order to fit into one line on a screen. Second, the stories described an everyday situation and an exchange between two characters who knew each other only casually (i.e., we avoided situations that presumed close relationships among interlocutors). Third, the first three sentences introduced the two characters and the situation. Fourth, the fourth and fifth sentences described the development of the situation that can be either positive (in the literal version) or negative (in the ironic version). These were the only two lines that could potentially change with respect to condition. Changes were designed to be as minimal as possible while keeping the stories sensible. Fifth, the sixth line was designed to be the target sentence. The length of all target sentences was between 10 and 12 syllables. Crucially, the target sentence (line 6) was exactly the same in both *Ironic* and *Literal* conditions and just the last word (e.g., “productive” in 1) allowed one to determine whether the sentence was ironic or not. Finally, the seventh line was an ordinary wrap-up conclusion of the story that makes sense for both the *Ironic* and *Literal* conditions (for an example see Table 1 and the Table S1).

**Table 1:** Examples of the four kinds of stimuli (translated from French).

Condition	Example
Ironic	Cynthia and Léa sing together in the same opera.On the night of the premiere they meet at the theatre.The show begins exactly on time.During their performance both ladies sing off key.After the show, Cynthia says to Léa:“Tonight we gave a superb performance.”As they take off their make-up they continue to discuss the show.Question: Do you think that the performance was in the morning?
Literal	Cynthia and Léa sing together in the same opera.On the night of the premiere they meet at the theatre.The show begins exactly on time.Both ladies sing beautifully and receive a rapturous round of applause.After the show, Cynthia says to Léa:“Tonight we gave a superb performance”As they take off their make-up they continue to discuss the show.Question: Do you think that the performance was in the morning?
Decoy	Mateo is relocating and has to move a very fragile and heavy mirror.He asks Paul for help.Paul makes himself available immediately.As soon as Paul lifts the mirror it breaks into a thousand pieces.Mateo says to Paul:"We have made a big mistake."A few days later, Mateo celebrates his move with his friends.Question: In your opinion, do Mateo and Damien move the mirror without problems?
Filler	Jeremy has promised to his kid to build him a cabin.He bought chestnut wood to build it.He works all the afternoon to finish it.In the end, the cabin is solid and well built.His kid is very happy and he tells him:“Come to play with my in the cabin.”They play all the weekend long in this new cabin.Question: Do you think that the cabin is well built?

Note. The examples from the *Ironic* and *Literal* conditions are drawn from the same framework; an individual participant would not receive both of these.

We aimed to prevent negative contexts from being cues to the presence of ironic remarks by introducing other stories in which a negative context (e.g., a bad performance) led to a plain, non-ironic utterance (e.g., “We will do better the next time.”). We refer to these stories as *decoys*. We created 30 decoys having the same structure as the ironic stories (a negative event that occurs early on in a 7-sentence story), except that the target sentence was banal. For example, the decoy story in Table 1 describes how one character drops a mirror, which leads the other character to remark “We have made a big mistake.” Like in the *Ironic* and *Literal* conditions, the target sentence in decoy stories is between 10 and 12 syllables (see also the Table S2 for further examples). We also designed 30 positive fillers in which a positive context was followed by a positive remark (for examples, see the Table S3).

Each participant read 30 ironic stories, 30 literal stories, 30 decoys and 30 positive fillers. For each participant, the 60 critical (non-decoy) stimuli were extracted randomly from a pool of 60 frameworks that could each be the basis of either an ironic or literal target sentence. The 30 decoys and the 30 positive fillers remained the same for each participant.

To verify that the stimuli used here were perceived as intended, a rating study was conducted on the 90 stories (2 from each of the frameworks plus the 30 decoys) with 42 participants (22 women) whose ages ranged from 19 to 38 (with a mean of 26) and who did not participate in the EEG study. Participants were asked to read each story and rate the extent to which the target sentence was ironic on a scale from 1 (not at all ironic) to 5 (very ironic). Ironic target sentences were rated as highly ironic (mean of 4.3), while literal sentences and the banal lines from the decoy stories were rated as low on the ironic scale (1.3 both of them). Repeated measure ANOVAs showed significant differences between (i) the Ironic and Literal conditions and (ii) the Ironic condition and Decoys (both at p<.001, corrected for multiple comparisons using the Tukey method). The comparison between the Literal condition and the Decoys was not significant (*p* = .1).

A yes/no comprehension question followed one third of the items (regardless of whether it was a critical or filler item). The question was about a detail in the story that made no reference to the target sentence. The purpose of the comprehension question was to ensure that the participants were paying attention to the stories. (See Table 1 for an example of all conditions and questions and the Appendix for further examples.).

### Procedure

**Figure 1:**
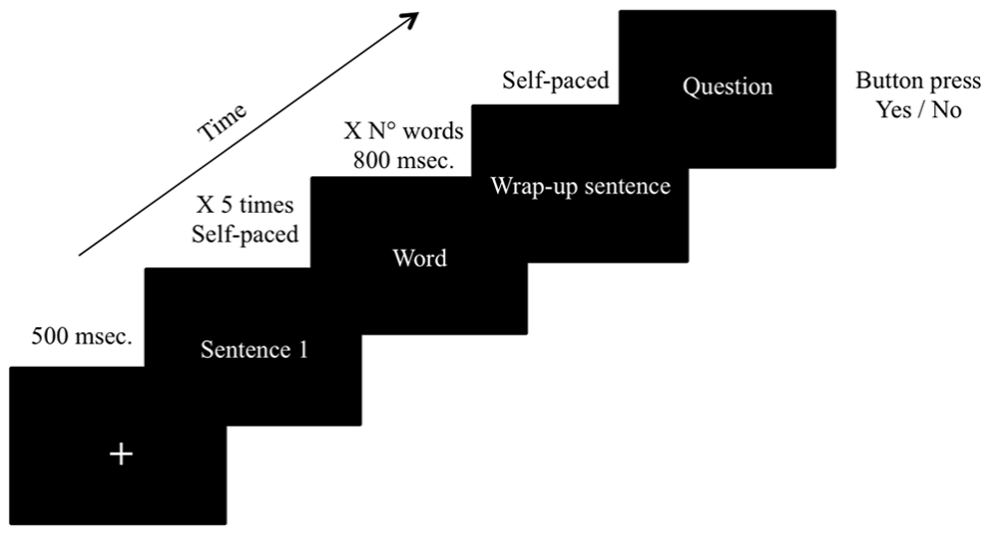
Experimental procedure.

Stimuli were prepared with Presentation 11.0 software (Neurobehavioral Systems, www.neurobs.com) and presented on a computer screen. Participants performed the experiment in four runs of 30 stories each. Each trial started with the presentation of a visual fixation mark (a white central cross). The participant read the stories line by line (i.e., sentence by sentence) in a self-paced manner (i.e., each sentence remained on the screen until the participant pressed a key) The interval between the disappearance of a sentence and the presentation of the next one was 500 ms. One line of each story was presented word-by-word in the center of the screen and each word appeared for 800 ms. In both the *Ironic* and *Literal* conditions the target line was always presented word-by-word. On the contrary, in the other 60 stories the line presented word-by-word was chosen randomly (see Figure 1) in order to prevent word-by-word presentation from being a cue about the experiment’s purpose. After the last sentence (line 7) disappeared, the question was presented in one third of the trials and the participants pressed one of two buttons on a mouse (yes/no response) to answer it (see Figure 1). Variable periods of visual fixation (1000 ms.+between 1 and 1000 ms.) were added at the end of each trial. The presentation order of the stories was pseudo-randomized. This means that the number of ironic and literal stories, decoys and fillers was balanced among the sessions. Fifteen ironic stories, 15 literal stories, 15 decoys and 15 positive fillers were presented in each half of the experiment. Each stimulus was displayed in a left-justified manner. Participants were instructed to read at a normal rate and to respond as accurately as possible to the questions. The experimental session began with 2 training trials, which did not include ironies. All told, a typical session lasted an hour (including breaks).

### Electroencephalogram (EEG) Recording

EEG data were recorded using BrainAmp amplifiers (Brain Vision recorder software, Brain Products GmbH, Munich, Germany). EEG was recorded from 32 scalp sites using the international 10–20 system [54], with a forehead ground. Impedance was 10 kΩ or less at the start of the recording. All scalp sites were referenced to the left mastoid. Horizontal and vertical eye movements were monitored using electro-oculograms (EOG) with a bipolar recording from electrodes placed around the left eye. The signal was sampled at 500 Hz.

### Analysis

Both the ERP and TFA were conducted using ELAN-Pack software developed at INSERM U821 (Lyon, France) [55]. Trials contaminated by eye blinks or eye movements (threshold: ±75 µV) were not included in the analyses nor were trials that were affected by drifts (range value 150 µV and latency: 500 ms.). Data from 3 subjects were excluded from the analyses because noise and eye movements contaminated more than half of the trials in the target condition.

#### ERP analysis

ERP analysis consisted of averaging the EEG segments in synchronization with the onset of the last word of the sentence presented word-by-word over 1250 ms. (250 ms. of pre-onset and 1000 ms. post-onset). The signals were band-pass filtered (0.16–30 Hz) and a baseline correction was computed from the 250 ms. to the 50 ms. preceding the onset of the target word and a notch filter was applied (50 Hz).

Nine representative electrodes of the 10–20 system were chosen to define different scalp regions (frontal: F3, Fz and F4; central: C3, Cz and C4 and parietal: P3, Pz and P4). We ran multiple ANOVAs using repeated measures that included *Intended-interpretation* (*Ironic* or *Literal*) and two levels of Electrode Site: *Laterality* (Left, Midline and Right) and *Anterior–Posterior* location (Frontal, Central, Parietal) as within-subject factors. Relevant post hoc comparisons were computed with Tukey HSD tests. We concentrated the statistical analysis on the 300–500 ms. time window in order to test the presence of the N400 effect and on the 500–800 ms. time window for the P600.

#### Time frequency analysis

Task-induced modulations of power across time and frequency were obtained by standard time–frequency analysis (TFA) using wavelets [56] over 1250 ms. (250 ms. of pre-onset and 1000 ms. post-onset of the last word of the target sentence). A baseline correction was computed from an interval of 50 to 250 ms. preceding the onset of the target word. We conducted statistical analyses over three frequencies bands: theta (4–7 Hz), alpha (8–12 Hz) and gamma (>30 Hz). In the gamma band the analysis of the signal was band-pass filtered in multiple successive 5 Hz wide frequency bands (e.g., [31]–[35], [36]–[40]). To test for significant increases or decreases in a frequency band, we used a paired-sample Wilcoxon signed rank test followed by a false discovery rate (FDR) correction across all time samples. The FDR approach yields a corrected threshold for significance [57] (*p*<.05).

## Results

### ERP Results

**Figure 2:**
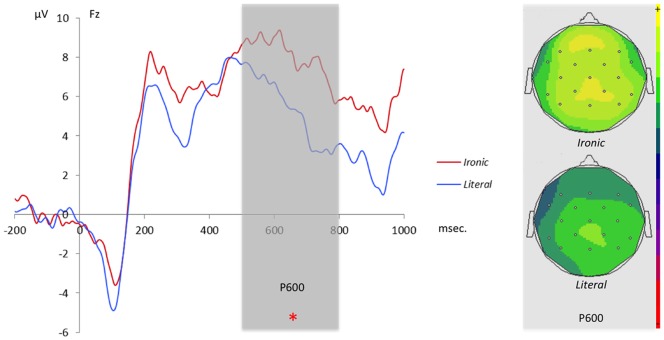
ERP waves for the*Ironic* (red line) and the *Literal* (blue line) conditions at Fz. The gray rectangle highlights the interval at which point the difference between the *Ironic* and the *Literal* condition is significant. On the right are the scalp distributions for both conditions at the peak of the P600 effect.

Figure 2 shows the time-course of the ERPs elicited by the *Ironic* and the *Literal* conditions at Cz. A repeated measures ANOVA on the 300–500 ms. time window showed a main effect for *Laterality* [*F*(2, 32) = 4.43, *p*<.05], *Anterior-Posterior* [*F*(2, 32) = 3.91, *p*<.05] as well as a significant interaction between the two variables [*F*(4, 64) = 2.62, *p*<.05]. However, the statistical analysis showed no significant difference between the *Ironic* and the *Literal* conditions [*F*(1, 16) = .03, *p*>.8] nor any interaction between the variable *Intended-interpretation* (*Ironic*/*Literal*) and the other variables. Therefore, our results do not reveal an N400 effect linked to Irony (see Appendix S1 and Figure S1 for a further analysis on a time window compatible with the N400). With respect to the 500–800 ms. time window, a repeated measure ANOVA revealed a main effect of *Laterality* [*F*(2, 32) = 11.23, *p*<.001], a marginally significant effect of the variable *Intended-interpretation* [*F*(1, 16) = 3.61, *p* = .075] and a significant interaction between *Intended-interpretation* and *Anterior-Posterior* [*F*(2, 32) = 5.84, *p*<.01]. A Tukey HSD test revealed that the difference between the *Ironic* and *Literal* conditions was due to a positive enhancement of the ERPs wave in the *Ironic* condition which were superior in the frontal sites. Considering the polarity and the shape of the ERP wave for the *Ironic* condition, we consider this difference in enhancement a P600 effect (see Figure 2).

### TFA Results

**Figure 3:**
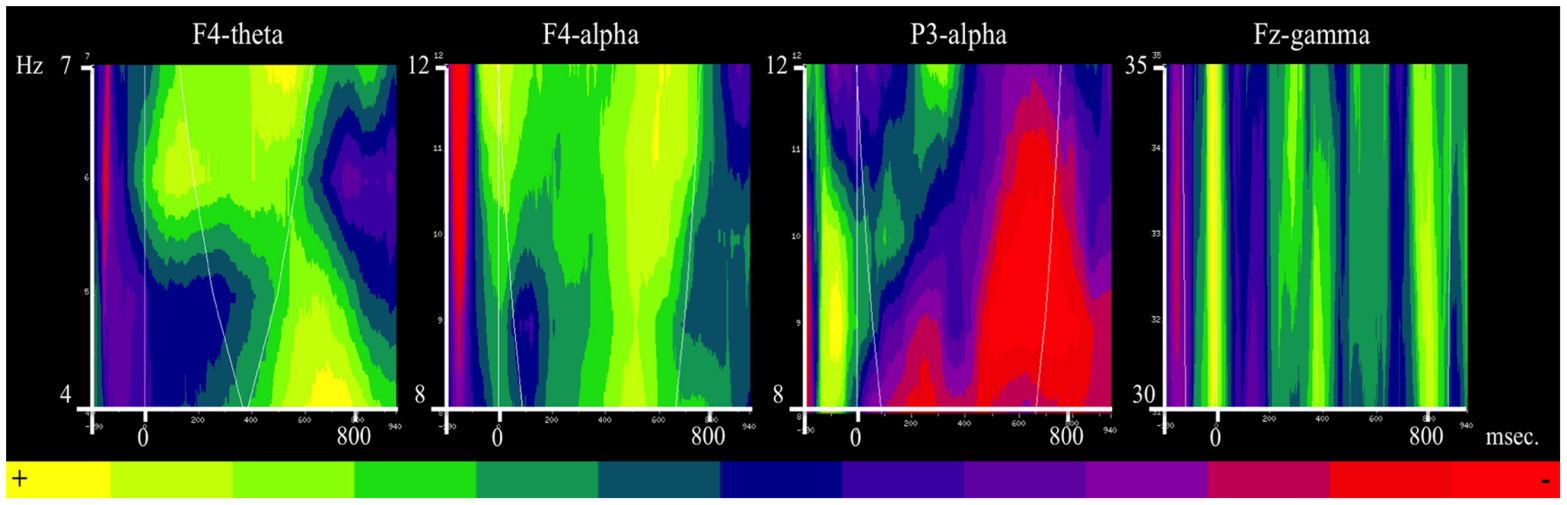
Time frequencies analysis. From the left: Z value for the contrast *Ironic*/*Literal* in the theta band at F4; Z value for the contrast *Ironic*/*Literal* in the alpha band at F4; Z value for the contrast *Ironic*/*Literal* in the alpha band at P3 and Z value for the contrast *Ironic*/*Literal* in the gamma band at Fz.

**Figure 4:**
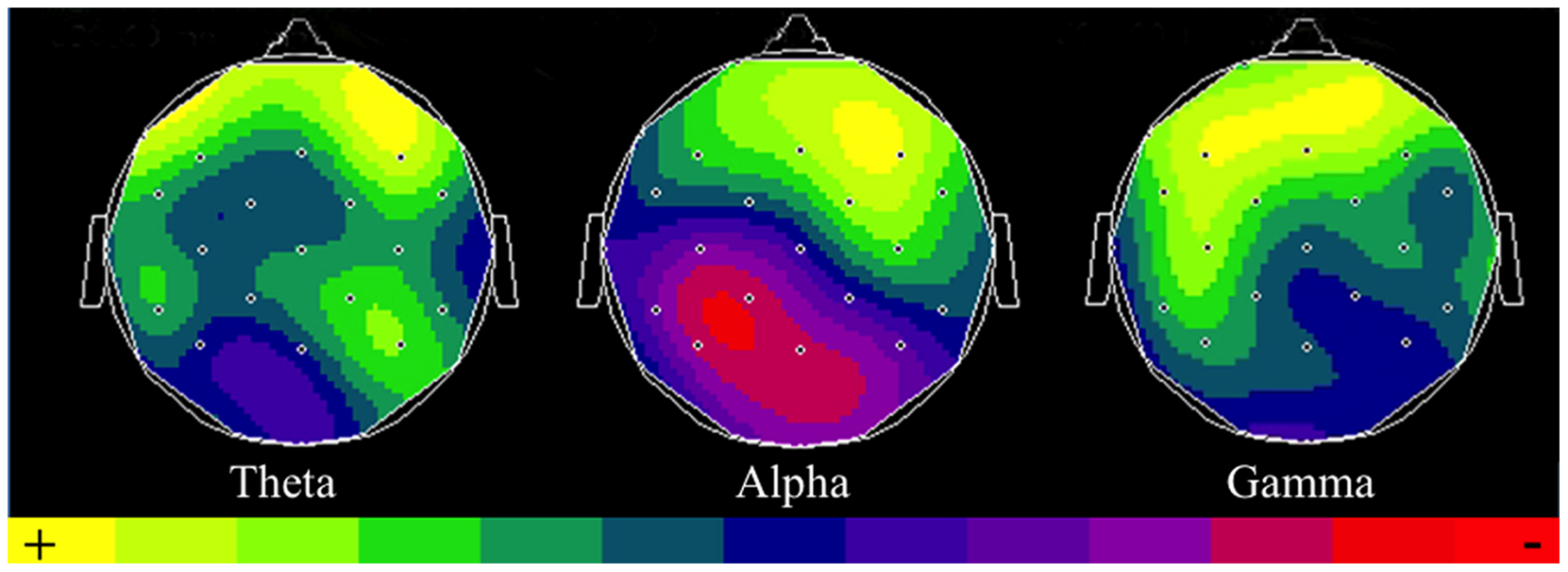
Scalp distribution of the different frequency bands at the significant time-windows. From the left: scalp distribution of theta waves (Z value for the contrast *Ironic*/*Literal*); scalp distribution of alpha waves (Z value for the contrast *Ironic*/*Literal*) and scalp distribution of gamma waves (Z value for the contrast *Ironic*/*Literal*).

Visual inspection of the spectrum of frequencies suggested the presence of an increase of power both in the theta band (4–7 Hz) and in the gamma band (30–90 Hz) during the *Ironic* condition. In addition, the power in the alpha band (8–12 Hz) seemed to increase in the frontal areas and to decrease in the parietal regions in this condition. The statistical analysis revealed that the difference of power in the theta band between the *Ironic* and the *Literal* conditions is significant between 500 and 700 ms., especially in the right frontal regions of the scalp (*Z* = 2.4; *p*<.05). In the alpha band, comparisons between the *Ironic* and *Literal* conditions were significantly different between (a) 400 and 700 ms. in the right frontal part of the scalp (*Z* = 2.6; *p*<.05) and between (b) 550 and 700 ms. in the left parietal areas (*Z* = − 1.7; *p*<.05). However, the variation (a) indicates an increase of power in the alpha band while the variation (b) reveals a decrease of power in the same band. The statistical analysis also showed a significant increase of power in the early range (31–35 Hz) of the gamma band between 280 and 400 ms. in the frontal areas of the scalp (*Z* = 2.4; *p*<.05) (see Figure 3 and 4). In the next section, we describe what these effects mean in cognitive terms.

## Discussion

Understanding a sentence is a complex act and much beyond syntax and semantics is necessary in order to grasp its intended meaning. The present study was designed to investigate those operations that can be best described as pragmatic during the comprehension of ironic remarks. We focused on irony because it represents a clear case in which the linguistic code underdetermines the speaker’s meaning and where pragmatic inferences are called on in order to fill the gap between the linguistic code of the sentence and its interpretation. We therefore investigated both ERPs and TFA in order to shed some light on this classic pragmatic form.

The ERP analysis focused on the N400 and the P600 because the literature shows that these two components are likely to be highly sensitive to pragmatic inference making. A classic view that relies primarily on decoding (e.g. an SPM type of account) would lead one to expect an N400 effect for the contrast *Ironic*>*Literal* because the literal meaning of an ironic remark is banally false, much like the sentence in (3b), “The Dutch trains are *white* and very crowded” which did indeed to lead to increased N400’s. In our case, the *Ironic*>*Literal* contrast did not amplify the N400 component, which is in line with results from the most recent ERP studies on irony processing [10], [11].

What then distinguishes (3b) from the ironic interpretation of (1)? In (3b), the violation of world knowledge prevents the sentence from being integrated into the context (i.e., the subject's representation of the state of affairs), while the surface structure of (1) – when preceded by an appropriate context – is simply part and parcel of an irony. Whereas the semantic violation in (3b) arguably interferes with processing, because it does not provide any obvious interpretational recourse, the apparent inconsistency between the context and the surface meaning of the target sentence in (1) only engages the listener to go beyond the linguistic code so as to look for a coherent interpretation (see [11] for a similar explanation). Although caution is called for in interpreting a null result, the absence of a N400 effect suggests that an inconsistency at the surface level is not in itself critical to irony processing.

Utterances in the *Ironic* condition – when compared to the *Literal* condition – elicited strong positive enhancements of ERP curves from 500 ms. onward. The shape and latency of the ERP curves allow us to consider this enhancement a P600 effect. As previously mentioned, it is hard to provide a functional interpretation of the P600, but Regel and colleagues [11] have considered three accounts for it. First, it can be assumed that the P600 is modulated by the predictability of the stimuli and by the experimental task (see for example, [58], [59]). Second, the P600 might index the processing of emotional arousal as caused by the ironic stimuli because it has been found that arousal (through pictures) and emotions (through words) can elicit an increase of the P600 in comparison with neutral controls (see e.g., [59]–[61]). Third, P600 modulation may reflect the processing of pragmatic inferences and, critically, the “reintegration of semantic meaning with extralinguistic information” [62].

Regel et al. [11] reject the first possibility due to arguments concerning the structure of their studies. We come to the same conclusion with respect to our own study and for the following two reasons. One is that the presence of decoys prevents negative contexts from being reliable cues to ironic remarks. The other is that the task consists of answering an easy comprehension question that never concerned the target line and it was present only in one third of the trials. Therefore, we are confident that the enhancement of the P600 is not due to our experiment’s design. We also agree with Regel et al. with respect to their rejection of the second possibility concerning the P600. As Regel et al. indicated with respect to their study, we are not convinced that the data in our study can be due to emotional arousal because the *Ironic*>*Literal* contrast does not compare an emotionally-charged remark with a neutral utterance. The target sentence, which is the same in both the *Ironic* and the *Literal* conditions, expresses an evaluation in both cases and arguably prompts comparable levels of arousal.

We are thus sympathetic with Regel et al.’s [11] proposal that the P600 reflects the processing of pragmatic inferences, we are just more circumspect with respect to their “semantic-extralinguistic” dichotomy. While we agree that previous studies have shown that the P600 reflects integration processes across a wide range of situations that can be characterized with this dichotomy (e.g., [62]) it strikes us as problematic for two reasons. First, it does not clearly address borderline cases. For example, it is not clear how the dichotomy can characterize Nieuwland and Van Berkum’s [20] finding showing that an apparent semantic violation (consider “… a woman talking to a *suitcase*”), which would be expected to prompt a classic N400, elicits a P600 when it is embedded in a discourse related to the anomalous noun (e.g., a story about traveling). The upshot is that the same sentence (the same linguistic unit) can be representative of either side of the dichotomy as a function of slight changes to context. Second, the semantic-extralinguistic distinction seems too general. That is, the P600 also emerges when listeners integrate syntactic and semantic information (e.g., [63], [64]) and this strikes us as much less *extra*linguistic than the other cases.

In a review of the N400 and P600 components, Gina Kuperberg [65] proposed that language processing engages at least two routes to utterance comprehension. The first is a semantic memory-based system that compares lexical information about the incoming words with information that is already stored in semantic memory; it is possible that the N400 reflects the work of such a system. The second route is a combinatorial process that integrates words that build up the propositional meaning on the basis of multiple constraints. She proposes that the P600 reflects continued combinatorial analysis, which ultimately determines the final interpretation of the sentence. Though her approach is centered on morphosyntactic and thematic–semantic constraints, the last line of the paper seems open to a broader interpretation:

*The idea that there are multiple distinct but interactive processing streams underlying comprehension helps explain how, on the one hand, we make maximal use of what we have encountered again and again in the real world, and yet how, on the other hand, we are able to compute unusual relationships between people, objects and actions to understand novel events. The balanced operation of these distinct brain systems – one that links incoming semantic information with existing information stored in semantic memory, and another that combines relationships between people, objects and actions to construct new meaning – allows for comprehension that is both efficient and yet adaptive. ([65], p. 45).*

Irony processing clearly requires one to construct a new meaning that goes beyond the lexical meanings of the incoming words. As we have already underlined, the addressee has to go beyond the linguistic code and integrate contextual information in order to grasp the complete meaning of an ironic utterance. Therefore, a P600 whose magnitude increases during the *Ironic* condition arguably indexes the continued combinatory analysis among different cognitive resources that lead to the interpretation of the ironic remark. In support of this account are studies that contrast false belief and true belief stories and likewise show an enhancement of a late positive slow wave (e.g., [66]–[69]). In conclusion, we argue that the enhancement of the P600 in the *Ironic*>*Literal* contrast reflects the integration between the linguistic stimulus and contextual information. However, as we indicated earlier, we were determined to better characterize these integration processes through time frequency analysis and this is what we turn to now.

The TFA shows an increase in power in the low gamma band during the *Ironic* condition when compared to the *Literal* one. Assuming that this enhancement of power in the gamma band indexes the integration operations that are necessary to grasp the complete meaning of an ironic utterance, this is an intriguing finding because the increase of power is significant early (in the 280–400 ms. time window). This indicates that integration operations during irony processing start well before the latency associated with the P600. This is a significant finding because classic views of language processing consider the integration between the linguistic code and the contextual information as one of the latter steps in the comprehension of an utterance (e.g., [1], [43]). While studies such as [15]–[17] have linked pragmatic aspects of language earlier to the N400, the N400 seems to intrinsically be an index of a mismatch between different streams of information e.g. the semantic meaning of a word and one’s world knowledge (e.g., the white Dutch trains in [39]) or the semantic meaning and the discourse context (e.g., the salted peanut in [15]). In contrast, the synchronization in the gamma band is arguably a more precise index of the integration between the linguistic code and the contextual information and it reveals that pragmatic inferences play a role in language comprehension very early on in processing, at least in the case of irony.

As we indicated earlier, pragmatic inference-making critically requires one to access the speaker’s intentions and this process involves cognitive resources that likely pertain to the domain of social cognition. There is already quite a bit of evidence that the gamma band can index social dimensions of communication (e.g., [42], [70]). Given our previous findings [7] – showing that linguistic and ToM networks interact in irony processing – we suggest that an increase of power in the gamma band might reflect the engagement of social cognitive processes that allow one to go beyond the linguistic code.

In line with previous studies on theta band (e.g., [32], [71]) we propose that the increase of power in the theta band reflects memory load during irony processing while, as has been pointed out in the Introduction, oscillation in the alpha band likely reflects the allocation of cognitive resources. We predicted that the integration of different streams of information is a critical and effortful aspect of irony processing and the oscillatory pattern in the alpha band ought to reveal that. The present study indeed reports a pattern of desynchronization and of specular synchronization of the alpha waves during roughly the same time-window as the P600. This seems to support our hypothesis.

### Contributions to the Debate on Irony Processing

A majority of studies on irony processing are focused on ongoing debates that pivot around the Standard Pragmatic Model, the Direct Access View and the Graded Salience Hypothesis. These theories are arguably focused on the alleged priority of a literal interpretation in an ironic remark while ignoring the cognitive mechanisms that are behind the comprehension of ironies; nevertheless, those accounts propose interesting predictions on irony processing and any researcher needs to face up to them.

The present work clearly supports accounts of irony understanding that reveal its complex and effortful nature; it extensively engages cognitive resources and it is demanding in terms of integration operations, as is reflected by both the P600 effect and the increase of power in the gamma band. Therefore, the data do not support the Direct Access View and they seem to be more compatible with either the SPM or the Graded Salience Hypothesis. However, the synchronization in the gamma band between 280 and 400 ms. seems to reveal that integration operations take place early in the comprehension process; this result cannot be easily accounted for by either of these two accounts because both assume that the integration between lexical aspects and contextual information occurs as part of a later-occurring second step. Therefore, the present study as well as Regel et al.’s [11] call for a revision of the dominant psycholinguistic models of irony processing.

A more recently developed position, called the Constraint-Satisfaction Approach [51], [72]–[74], seems to fit well with the present data. Following this proposal, all available information – such as lexical entries and contextual cues – are integrated as soon as they are relevant in order to derive a coherent representation of the speaker’s intent. Therefore, an ironic interpretation of the utterance is considered as soon as there is sufficient evidence that it might be supported. The constraint-satisfaction approach is compatible with results revealing extra effort in irony processing, (the increase of the P600) as well as those showing that integration operations take place early on, e.g., the increase of power in the gamma band between 280 and 400 ms. However, further studies are necessary to determine which constraints are being satisfied.

### Conclusion

The results we presented support the hypothesis that pragmatic inferences basically require the addressee to integrate the linguistic stimulus with the information that she can extract from the context. The increase in magnitude of the P600 during the *Ironic* condition when compared to the *Literal* one reflects effort as one integrates the ironic utterance with the preceding context, while the increase of power in the gamma band for the contrast *Ironic*>*Literal* reveals that the integration between different streams of information takes place early in the comprehension process. While our reading of the TFA data is generally in line with the interpretation provided by Bastiaansen and colleagues [24] these results are newsworthy because we have extended the analysis so that it considers a pragmatic dimension. We have focused our investigation on irony because it is a prototypical pragmatic case that encourages a listener to go beyond the linguistic code in order to grasp the utterance’s intended meaning. We anticipate that the reported findings, coming from several angles (i.e., P600, the pattern of oscillations in the gamma band), as well as the interpretation that we provide for them can eventually be applied to pragmatic inference-making more generally.

## Supporting Information

Figure S1 **ERP waves for the Ironic (red line) and the Literal (blue line) conditions at Pz.**
(TIF)

Table S1 **Examples of ironic and literal stories.**
(DOC)

Table S2 **Examples of decoys.**
(DOC)

Table S3 **Examples of positive fillers.**
(DOC)

Appendix S1 **Analysis in the N400 time window.**
(DOC)
