# Supplementary figures and images for: What’s behind a P600? Integration Operations during Irony Processing
Source: PLoS One. 2013 Jun 24;8(6):e66839. doi: 10.1371/journal.pone.0066839 (PMC3691266; doi:10.1371/journal.pone.0066839)

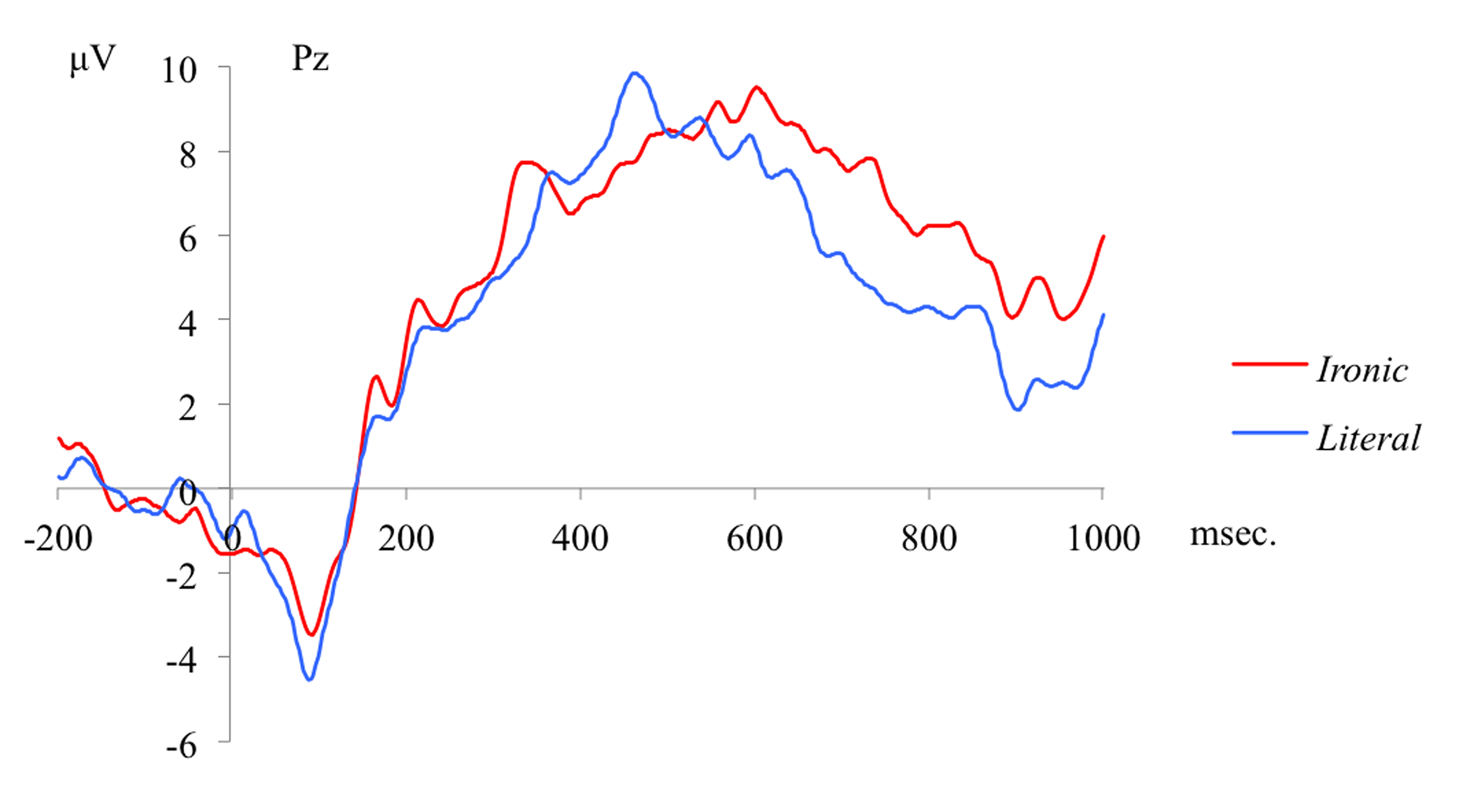

Supplement: Figure S1 — ERP waves for the Ironic (red line) and the Literal (blue line) conditions at Pz. (TIF) [file pone.0066839.s001.tif]
