# Supplementary material for: What’s behind a P600? Integration Operations during Irony Processing
Source: PLoS One. 2013 Jun 24;8(6):e66839. doi: 10.1371/journal.pone.0066839 (PMC3691266; doi:10.1371/journal.pone.0066839)
Supplement: Table S1 — Examples of ironic and literal stories. (DOC) [file pone.0066839.s002.doc]

Table S1

Examples of ironic and literal stories

| Condition | French (as presented) | English translations |
| --- | --- | --- |
| Ironic | Cynthia et Léa chantent dans le même opéra.  Le soir de la première, elles se retrouvent au théâtre.  Le spectacle commence pile à l’heure.  Durant la représentation, elles font beaucoup de fausses notes.  Après le spectacle, Cynthia dit à Léa :  « Ce soir on a fait une performance magistrale. »  Tandis qu’elles se démaquillent, les deux filles continuent à parler du spectacle.  Question : À votre avis, est-ce que la performance est le matin ? | Cynthia and Léa sing together in the same opera.  On the night of the premiere they meet at the theatre.  The show begins exactly on time.  During their performance they often sing off key.  After the show, Cynthia says to Léa:  “Tonight we gave a superb performance.”  As they take off their make-up they continue to discuss the show.  Question: In your opinion, do you think that the performance was in the morning? |
| Literal | Cynthia et Léa chantent dans le même opéra.  Le soir de la première, elles se retrouvent au théâtre.  Le spectacle commence pile à l’heure.  La représentation est excellente et les chanteurs sont longuement applaudis.  Après le spectacle, Cynthia dit à Léa :  « Ce soir on a fait une performance magistrale. »  Tandis qu’elles se démaquillent, les deux filles continuent à parler du spectacle.  Question : À votre avis, est-ce que la performance est le matin ? | Cynthia and Léa sing together in the same opera.  On the night of the premiere they meet at the theatre.  The show begins exactly on time.  The show was excellent and the singers were given a long applause.  After the show, Cynthia says to Léa:  “Tonight we gave a superb performance.”  As they take off their make-up they continue to discuss the show.  Question: In your opinion, do you think that the performance was in the morning? |
| Ironic | Lors d’un dîner, Patrick parle à son collègue Pascal de sa fondation contre l’anorexie.  Il explique à Pascal sa nouvelle idée.  Il lui dit qu’il va lancer une nouvelle campagne de financement.  Quelques mois après ils se retrouvent pour évaluer les résultats décevants de la campagne.  Les gens ont donné beaucoup moins cette fois-ci et Pascal dit à Patrick :  « Avec cette campagne on a fait un grand coup. »  Patrick et Pascal commencent à penser aux nouvelles activités pour la fondation.    Question : A votre avis, est-ce que Pascal et Patrick sont collègues ? | While at dinner, Patrick talks to his colleague Pascal about his foundation to combat anorexia.  He explains to Patrick his new idea.  He tells him that he is going to begin a new fundraising campaign.  Several months later, they meet again to evaluate the disappointing results of the campaign.  People gave much less this time and Pascal says to Patrick:  “This campaign has really been a hit.”  Patrick and Pascal start to think of new activities for the foundation.  Question: In your opinion, do you think Pascal and Patrick are colleagues? |
| Literal | Lors d’un dîner, Patrick parle à son collègue Pascal de sa fondation contre l’anorexie.  Il explique à Pascal sa nouvelle idée.  Il lui dit qu’il va lancer une nouvelle campagne de financement.  Quelques mois après ils se retrouvent pour évaluer les excellents résultats de la campagne.  Les gens ont donné beaucoup plus que les années précédentes et Pascal dit à Patrick :  « Avec cette campagne on a fait un grand coup ! »  Patrick et Pascal commencent à penser aux nouvelles activités pour la fondation.    Question : A votre avis, est-ce que Pascal et Patrick sont collègues ? | While at dinner, Patrick talks to his colleague Pascal about his foundation to combat anorexia.  He explains to Patrick his new idea.  He tells him that he is going to begin a new fundraising campaign.  Several months later, they meet again to evaluate the excellent results of the campaign.  People gave much more than in previous years and Pascal says to Patrick:  “This campaign has really been a hit!”  Patrick and Pascal start to think of new activities for the foundation.  Question: In your opinion, do you think Pascal and Patrick are colleagues? |
| Ironic | Luc parle de ses investissements à Alfred qui est courtier en bourse.  Luc voudrait investir son argent dans les actions d’une petite entreprise.  Alfred lui explique les avantages et les inconvénients.  Luc achète les actions mais un mois plus tard leur valeur a diminué de moitié.  Lors de la réunion suivante, Luc en reparle à Alfred et dit :  « C’est ce qui s’appelle un investissement rentable. »  Pendant qu’ils parlent, les nouveaux cours de la bourse s’affichent sur le portable de Luc.  Question : A votre avis, est-ce que Luc veut investir dans une grande entreprise ? | Luc talks about an investment with Alfred, who is a stockbroker.  Luc is interested in buying shares of a small company.  Alfred describes the advantages and inconveniences of such an investment.  Luc buys the stocks, but one month later their value has dropped by half.  At the next meeting, Luc talks about it with Alfred again and says:  "This is what’s called a worthwhile investment."  As they are talking, Luc’s laptop displays new stock quotes.  Question: In your opinion, does Luc want to invest in a big company? |
| Literal | Luc parle de ses investissements à Alfred qui est courtier en bourse.  Luc voudrait investir son argent dans les actions d’une petite entreprise.  Alfred lui explique les avantages et les inconvénients.  Un mois plus tard, le cours des actions de Luc a déjà doublé.  Au cours d’un déjeuner, il en reparle à Alfred :  « C’est ce qui s’appelle un investissement rentable. »  Pendant qu’ils parlent, les nouveaux cours de la bourse s’affichent sur le portable de Luc.  Question : A votre avis, est-ce que Luc veut investir dans une grande entreprise ? | Luc talks about an investment with Alfred, who is a stockbroker.  Luc is interested in buying shares of a small company.  Alfred describes the advantages and inconveniences of such an investment.  One month later their value, Luc’s stocks have already doubled.  Over a lunch, Luc talks about it again with Alfred:  "This is what’s called a worthwhile investment."  As they are talking, Luc’s laptop displays new stock quotes.  Question: In your opinion, does Luc want to invest in a big company? |
| Ironic | Clara et Isabelle doivent décider quel film aller voir au cinéma.  Elles remarquent l’affiche d’un film dans la rue.  Elles ne le connaissent pas mais décident d’aller le voir.  Les deux amies achètent les billets et des pop-corn.  Le film se révèle être banal et très ennuyeux, Clara dit alors à Isabelle :  « Nous sommes allées voir un film formidable. »  Elles sortent de la salle et vont s’acheter une glace.  Question : A votre avis, est-ce que Clara et Isabelle vont s’acheter une glace ? | Clara and Isabelle must decide which film to see at the cinema.  They see a poster for a film outside.  They aren’t familiar with it but they decide to go see it.  The two friends buy tickets and popcorn.  The film turns out to be banal and very boring, so Clara says to Isabelle:  “We went to see a wonderful film.”  They leave the theater and go buy an ice cream.  Question: In your opinion, do you think Clara and Isabelle went to buy an ice cream? |
| Literal | Clara et Isabelle doivent décider quel film aller voir au cinéma.  Elles remarquent l’affiche d’un film dans la rue.  Elles ne le connaissent pas mais décident d’aller le voir.  Les deux amies achètent les billets et des pop-corn.  Le film se révèle être excitant et surprenant, Clara dit alors à Isabelle :  « Nous sommes allées voir un film formidable. »  Elles sortent de la salle et vont s’acheter une glace.  Question : A votre avis, est-ce que Clara et Isabelle vont s’acheter une glace ? | Clara and Isabelle must decide which film to see at the cinema.  They see a poster for a film outside.  They aren’t familiar with it but they decide to go see it.  The two friends buy tickets and popcorn.  The film turns out to be exciting and surprising, so Clara says to Isabelle:  “We went to see a wonderful film.”  They leave the theater and go buy an ice cream.  Question: In your opinion, do you think Clara and Isabelle went to buy an ice cream? |
