# Supplementary material for: What’s behind a P600? Integration Operations during Irony Processing
Source: PLoS One. 2013 Jun 24;8(6):e66839. doi: 10.1371/journal.pone.0066839 (PMC3691266; doi:10.1371/journal.pone.0066839)
Supplement: Table S2 — Examples of decoys. (DOC) [file pone.0066839.s003.doc]

Table S2: Examples of decoys

| French (as presented) | English translation |
| --- | --- |
| Matéo déménage et doit déplacer un miroir lourd et très fragile.  Il demande à Paul de l’aider.  Paul est disponible tout de suite.  A peine a-t-il soulevé le miroir que ce dernier se brise en mille morceaux.  Matéo dit à Paul:  « On a fait une grosse bêtise. »  Quelques jours plus tard, Matéo fête son emménagement avec des amis.  Question: A votre avis, est-ce que Mateo et Paul ont déménagé le miroir sans problème? | Matéo is relocating and has to move a very fragile and heavy mirror.  He asks Paul for help.  Paul makes himself available immediately.  As soon as Paul lifts the mirror it breaks into a thousand pieces.  Mateo says to Paul:  "We have made a big mistake."  A few days later, Mateo celebrates his move with his friends.  Question: In your opinion, do Matéo and Damien move the mirror without problems? |
| Damien et Myriam vont faire les soldes.  Damien n’a pas une idée trop claire sur ce qu’il veut acheter.  Myriam lui propose de tester différents magasins.  En ressortant d’une cabine d’essayage, Damien est vêtu d’une manière très extravagante.  En voyant le résultat, il dit à Myriam :  « Je n'aime pas ces habits. »  Ils décident alors de passer au prochain magasin.  Question: A votre avis, est-ce que Damien et Myriam vont chercher des nouveaux vêtements ? | Damien and Myriam go shopping for clothes on sale.  Damien doesn’t have a very clear idea of what he wants to buy.  Myriam suggests that he try different stores.  Damien comes out of one dressing room clothed in a very extravagant manner.  Seeing himself in the mirror, he says to Myriam:  “I don’t like these clothes.”  They decide to move on to the next store.  Question: In your opinion, do you think Damien and Myriam are shopping for new clothes? |
| Valérie organise une surprise pour l’anniversaire d’une copine.  Elle demande à Romain de l’aider.  Romain est tout à fait d’accord.  Malheureusement, la copine en question découvre le secret une semaine avant la fête.  Valérie dit à Romain :  « L’effet de surprise est complètement raté. »  Cependant la fête a été un succès.  Question: A votre avis, est-ce que Valérie cherche à organiser une surprise ? | Valérie organizes a surprise party for a (female) friend’s birthday.  She asks Romain to help her.  Romain agrees.  Unfortunately, the friend in question discovers the secret a week before the party.  Valérie says to Romain:  “The surprise is completely ruined.”  Nonetheless the party was a success.  Question: In your opinion, do you think Valérie tried to organize a surprise party? |
| Hugo travaille dans une usine de voiture au service de la sécurité routière.  Joël vient le voir le jour des crashs tests.  Hugo lui montre les procédures pour les tests.  Ils réalisent alors un test avec un mannequin assis dans la voiture.  Le mannequin est complètement détruit à la fin du test et Hugo dit à Joël :  « Il y a sûrement eu une erreur quelque part. »  Hugo pense alors aux études faites avec les nouvelles normes de sécurité.  Question : A votre avis, est-ce que le mannequin est intact après le test ? | Hugo works at a car factory in the department of road safety.  Joel comes to see him on the day of the crash tests.  Hugo shows him the testing procedures.  They run a test with a dummy seated in the car.  The dummy is completely destroyed at the end of the test and Hugo says to Joel:  "There must have been an error somewhere."  Then Hugo remembers the studies done using the new safety standards.  Question: In your opinion, is the dummy intact after the test? |
