# Supplementary material for: What’s behind a P600? Integration Operations during Irony Processing
Source: PLoS One. 2013 Jun 24;8(6):e66839. doi: 10.1371/journal.pone.0066839 (PMC3691266; doi:10.1371/journal.pone.0066839)
Supplement: Table S3 — Examples of positive fillers. (DOC) [file pone.0066839.s004.doc]

Table S3

Examples of positive fillers

| French (as presented) | English translation |
| --- | --- |
| Jérémy a promis à son fils de lui construire une cabane.  Il a acheté du bois de châtaignier pour la fabriquer.  Il travailla tout l’après-midi pour la bâtir.  Une fois terminée, la cabane est très solide et bien construite.  Son fils est très heureux et dit à son père :  « Viens jouer avec moi dans la cabane. »  Ils jouèrent tous les deux durant tout le week-end dans cette nouvelle cabane.  Question : A votre avis, est-ce que la cabane est bien construite ? | Jeremy has promised to his kid to build him a cabin.  He bought chestnut wood to build it.  He works all the afternoon to finish it.  In the end, the cabin is solid and well built.  His kid is very happy and he tells him:  “Come to play with my in the cabin.”  They play all the weekend long in this new cabin.  Question: Do you think that the cabin is well built? |
| Hubert a invité son collègue Michael qui est un grand amateur de vin.  Hubert sort une bonne bouteille.  Il demanda à Michael s’il veut goûter le vin en premier.  Le vin est extrêmement doux, une véritable merveille.  Durant le dîner Michael demande à Hubert :  « Est-ce que tu es collectionneur de grands vins ? »  La soirée fut très agréable pour toutes les personnes présentes à ce dîner.  Question : A votre avis, est-ce que Michael boit du vin pour la première fois ? | Hubert has invited his colleague Michael who is a wine expert.  Hubert open one of his bottles.  He asks to Michael if he wants to taste the wine as first.  The wine is extremely mellow, a real marvel.  During the dinner Michael asks to Hubert:  “Are you a collector of wine?”  The night has been enjoyable for all the people that participate to the dinner.  Question: Do you think that Michael drinks wine for the first time in his life? |
| Pauline a conseillé un restaurant à Sarah et Thomas.  Ils viennent à Lyon pour la première fois.  Pauline ajoute que le restaurant qu’elle leur propose est bon marche.  En effet, les plats sont délicieux et en plus ils ne sont pas chers.  Le lendemain, Sarah appelle Pauline et lui dit :  « Nous devrions aller ensemble dans ce restaurant. »  Les deux filles prirent alors rendez-vous le samedi suivant pour le diner.  Question : A votre avis, est-ce que Sarah a apprécié le restaurant ? | Pauline has suggested a restaurant to Sarah and Thomas.  They are going to Lyon for the first time.  Pauline adds that the restaurant she has suggested them is cheap.  In fact, the food is delicious and they are not expensive.  The day after, Sarah calls Pauline and tells her:  “We have to go together to that restaurant.”  The two girls take an appointment for a dinner Saturday after.  Question: Do you think that Sarah likes the restaurant? |
| Jonathan et Bastien partent à la plage.  Bastien prête sa crème solaire à Jonathan.  Il a acheté une crème nouvelle juste ce matin.  A la fin de la journée, Jonathan n’a pas pris de coup de soleil.  En rentrant, Jonathan dit à Bastien :  « Ta crème est d’une surprenante efficacité. »  Après la plage les deux amis vont manger une pizza dans le centre-ville.  Question : A votre avis, est-ce que la crème était inutile? | Jonathan and Bastien are going to the beach.  Bastien leads his sunscreen to Jonathan.  He has bought a new sunscreen just that morning.  In the end of the day, Jonathan did not sunburn.  While they are going back, Jonathan tells to Bastien:  “Your sunscreen is really effective.”  After the beach, the two friends go to eat a pizza downtown.  Question: Do you think that the sunscreen has not been useful? |
