# Supplementary material for: What’s behind a P600? Integration Operations during Irony Processing
Source: PLoS One. 2013 Jun 24;8(6):e66839. doi: 10.1371/journal.pone.0066839 (PMC3691266; doi:10.1371/journal.pone.0066839)
Supplement: Appendix S1 — Analysis in the N400 time window. (DOC) [file pone.0066839.s005.doc]

Appendix S1

In order to ensure that there is no significant effect in the N400 time window, we also conducted an analysis with respect to the latency that, following a visual inspection of the parietal sites (where, usually, the peak of the N400 is recorded), should most likely reveal an enhancement of the N400 component in the *Ironic* condition when it is compared to the *Literal* one. The repeated measure ANOVA on the time window 360 – 510 msec. showed a main effect of both *Laterality* [*F*(2, 32) = 4.55, *p* < .05] and *Anterior-Posterior* [*F*(2, 32) = 3.44, *p* < .05] as a significant interaction between the two variables [*F*(4, 64) = 2.73, *p* < .05]. However, the statistical analysis showed no significant difference between the *Ironic* and the *Literal* conditions [*F*(1, 16) = .23, *p* > .6] nor any interaction between the variable *Intended-interpretation* (*Ironic* / *Literal*) and the other variables. Therefore, we are confident that our results do not reveal a N400 effect (see Figure S1).
